# Supplementary figures and images for: Genetic diversity and evolutionary convergence of cryptic SARS- CoV-2 lineages detected via wastewater sequencing
Source: PLoS Pathog. 2022 Oct 14;18(10):e1010636. doi: 10.1371/journal.ppat.1010636 (PMC9604950; doi:10.1371/journal.ppat.1010636)

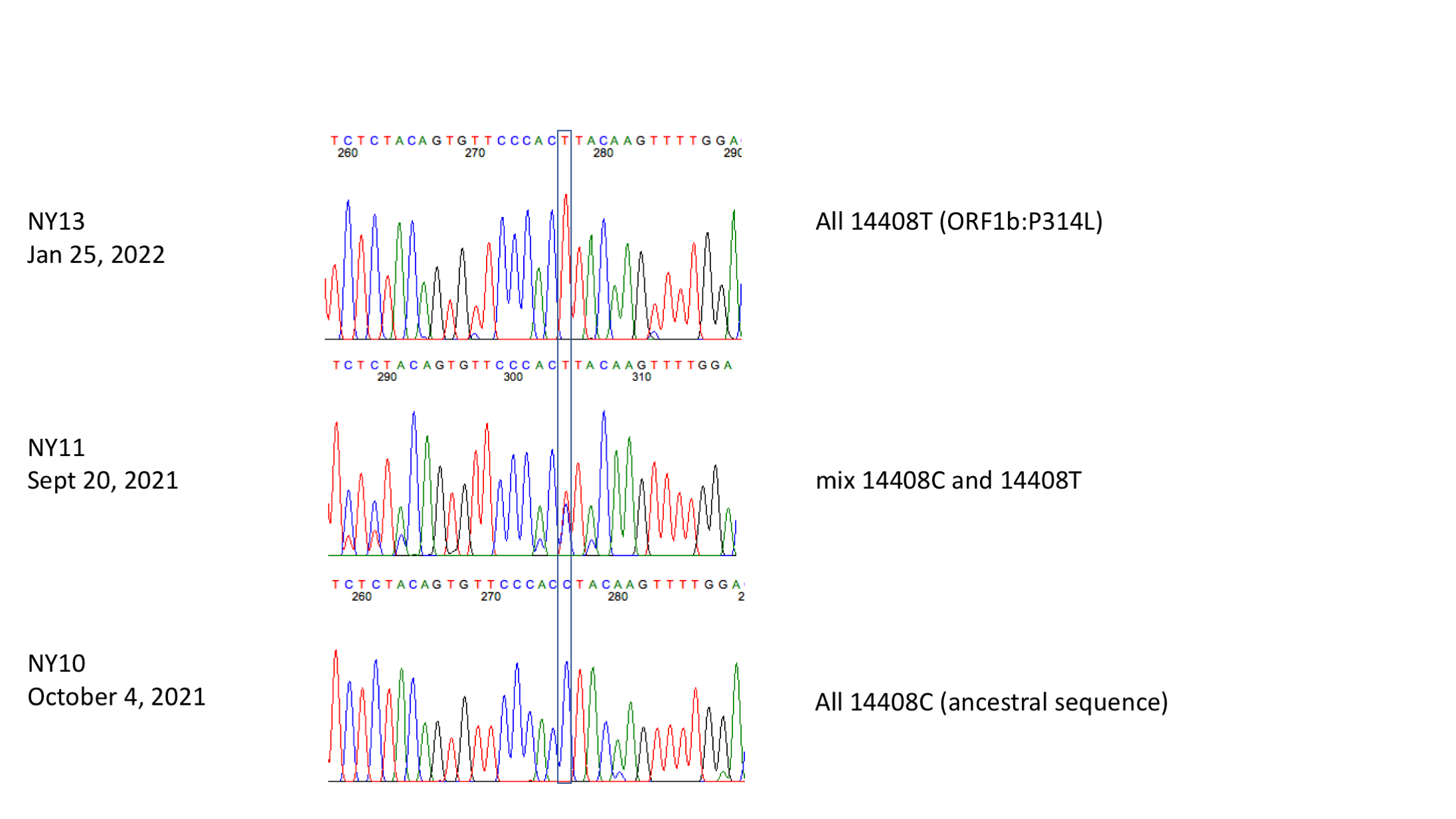

Supplement: S1 Fig — (TIFF) [file ppat.1010636.s001.tiff]
