# Supplementary material for: Genetic diversity and evolutionary convergence of cryptic SARS- CoV-2 lineages detected via wastewater sequencing
Source: PLoS Pathog. 2022 Oct 14;18(10):e1010636. doi: 10.1371/journal.ppat.1010636 (PMC9604950; doi:10.1371/journal.ppat.1010636)
Supplement: S1 Table — (DOCX) [file ppat.1010636.s002.docx]

**S1 Table. Prevalence in GISAID of common substitutions found in cryptic lineages.** Data reflects the number of sequences from humans deposited into GISAID by the indicated dates [[1]](https://www.zotero.org/google-docs/?lYZmi1).

| **Substitution** | **Location** | **Global Prevalence in Humans 11/1/21** | **Global Prevalence**  **3/15/22** |
| --- | --- | --- | --- |
| Total Sequences |  | 4,824,812 | 9,349,201 |
| G413R | NY10, NY14 | 147 | 239 |
| K417T | MO33, MO45, NY2, NY3, NY11, NY13, NY14,  CA | 108,537 | 119,127 |
| N439K | MO33, NY2, NY3 | 37,227 | 40,274 |
| N440K | MO33 | 9,154 | 1,652,112 |
| K444Δ | NY14 | 14 | 68 |
| K444S | NY2, NY11 | 21 | 26 |
| K444T | NY2, NY10, NY11, NY14 | 58 | 217 |
| V445Δ | NY11, NY14 | 24 | 84 |
| V445A | CA, NY3, NY13, NY14 | 298 | 557 |
| G446S | CA, NY2 | 472 | 1,336,425 |
| G446Δ | NY11 | 19 | 82 |
| G446D | NY3, NY13 | 75 | 224 |
| Y449R | NY2, NY3, NY11 | 0 | 0 |
| L452Q | NY2, NY3 | 10,498 | 12,137 |
| L452R | MO33, NY3, NY14 | 2,315,718 | 4,324,990 |
| Y453F | NY3 | 1,320 | 1,497 |
| F456L | NY2,NY3, NY10, NY14 | 259 | 736 |
| N460K | NY2, CA, MO33, MO45, NY3, NY10, NY11, NY13, NY14 | 76 | 242 |
| S477N | CA, NY3, NY10, NY14 | 71,960 | 2,164,897 |
| T478K | CA, MO45, NY2, NY3 | 2,249,016 | 6,340,479 |
| V483Δ | NY2 | 49 | 932 |
| E484Δ | NY3, NY11 | 31 | 912 |
| E484A | CA, MO45, NY2, NY10, NY11, NY13 | 551 | 2,087,453 |
| E484P | NY2 | 0 | 73 |
| E484V | NY3, NY10 | 104 | 1,610 |
| F486P | CA, NY3, NY10, NY11 | 2 | 3 |
| F486V | NY13, NY14 | 4 | 34 |
| F490Y | CA, NY2, NY10, NY11, NY14 | 120 | 163 |
| Q493R | NY3, NY14 | 261 | 2,083,669 |
| Q493K | MO33, MO45, NY10, NY13, NY14 | 152 | 835 |
| S494P | MO45, NY2, NY10 | 12,916 | 15,009 |
| Q498H | CA, MO45, NY2, NY11, NY14 | 36 | 57 |
| Q498R | NY13 | 91 | 2,007,408 |
| Q498Y | NY2, CA, NY3, NY10, NY11, NY14 | 0 | 13 |
| P499S | CA, NY14 | 216 | 353 |
| N501S | CA, NY10, NY11, NY14 | 166 | 663 |
| N501T | CA, MO33, NY2, NY3, NY10, NY11, NY14 | 4,742 | 5,639 |
| N501Y | NY11, NY13 | 1,325,387 | 3,389,688 |
| G504D | NY3, NY14 | 190 | 580 |
| Y505H | CA, NY2, NY3, NY11, NY13,  NY14 | 133 | 2,013,881t |
| H519N | NY10, NY11 | 13 | 31 |
| T572I | CA, NY2 | 16,610 | 26,948 |
| T572N | NY10, NY14 | 148 | 298 |

 References

1. Khare S, Gurry C, Freitas L, Schultz MB, Bach G, Diallo A, et al. GISAID’s Role in Pandemic Response. China CDC Wkly. 2021;3: 1049–1051. doi:10.46234/ccdcw2021.255
