# Supplementary material for: Genetic diversity and evolutionary convergence of cryptic SARS- CoV-2 lineages detected via wastewater sequencing
Source: PLoS Pathog. 2022 Oct 14;18(10):e1010636. doi: 10.1371/journal.ppat.1010636 (PMC9604950; doi:10.1371/journal.ppat.1010636)
Supplement: Attachment — Submitted filename: Response to reviewer comments .docx [file ppat.1010636.s013.docx]

We thank the reviewers for their thoughtful and constructive comments.

At the advice of reviewers, we have made several changes in the display of data in the figures. We believe these changes have significantly strengthened the manuscript. Those changes are outlined here and also mentioned below in the point-by-point responses:

1. The formula used for displaying cryptic lineage was done in a uniform and clearly defined way. Note: this resulted in slightly more stringent filtering of lineages in the figures. As a result, there were minor changes in some of the mutations that are displayed in the figures. However, the comprehensive list of lineages detected are now included in supplemental data.

2. The lineages are grouped by similarity (based on MAFFT alignment) rather than by date of detection.

3. The main variants of concern (VOC) and their prevalence are displayed in all figures.

4. The 'Omicron' mutations now incorporate BA.1, BA.2, and BA.5 mutations.

5. The displays now show both synonymous mutations and non-synonymous mutations.

**Summaries.**

**Reviewer 3 noted, " This is not entirely novel since the same authors have other similar manuscripts. The employed methodology allowed the researchers to monitor SARS-CoV-2 lineages circulating for months in different areas, which may be helpful. Authors claimed that their findings demonstrate that "SARS-COV-2 genetic diversity is greater than what is commonly observed through routine SARS-CoV-2 surveillance", which was already reported in other manuscripts. The authors speculate that one of the possible sources of the cryptic lineages observed in sewage, but never found in human cases, may have their origin from animal reservoirs. However, there is no substantial evidence to support this hypothesis."**

We respectfully assert that the findings from this manuscript significantly extend previous findings from our group and other groups. Specifically:

1. Cryptic lineages are not just a phenomenon of NYC wastewater and an artifact of their early, intense epidemic in early 2020, they have been identified across the country.

2. Cryptic lineages identified to date were derived from pre-VOC lineages, but they can be derived from other lineages (Alpha), making it likely that cryptic lineages templated on currently circulating VOC (ie., Omicron) will be found in the future.

3. The 'unique' mutations in the Omicron Spike RBD were all seen in cryptic lineages prior to Omicron's emergence, consistent with convergent evolution.

4. Cryptic lineages are not static but change over time within the same sewershed.

5. The Spike RBD from some cryptic lineages is more divergent than anything seen in human clinical samples.

**Major points.**

**Reviewer 1.**

**Have any controls being performed to make sure the sequencing is actually capturing viral RNA rather than DNA? Both New York City and parts of Missouri are home to major research universities that are performing experiments on SARS-CoV-2, including construction of plasmids encoding mutants of the SARS-CoV-2 spike. For instance, in New York City the Bieniasz lab at Rockefeller created a polymutant spike in VSV that has many RBD mutations.**

At the reviewer's suggestion we ran a non-RT reaction with several of the samples that we knew contained the cryptic lineages and the results were all negative. It was not possible to perform this with all of the samples because we have no remaining sample for most of the time points. Several of the sewersheds have no major research institutes. We have been in periodic with the Bieniasz lab and none of the sequences match those selected in their assays.

**Many PCR cycles (~50) are performed to amplify the RBD for sequencing. This creates two potential concerns: PCR chimerism (where different mutations from different molecules are scrambled due to recombination during the PCR reaction) and jackpot amplification of specific molecules. For the jackpot amplification, this can be largely corrected for by splitting the original sample into halves, performing the entire prep and sequencing separately, and then seeing if the same mutants are identified. It is somewhat unclear how systematically this was done: the methods say something about "sequences that appeared in only one sample... were discarded", but more elaboration on this point would be useful. For the PCR chimerism (recombination), it would be useful to mention this more prominently as a caveat particularly with respect to different sequences that have subsets of the same pool of mutations.**

**Overall, more description of the exact cutoffs used to call the variants, and the sensitivity of the results to these cutoffs, should be included in the main text Results.**

We added an explicit reference in the results to the software pipeline that we developed for removing PCR chimeras.

We did not generally do split samples, but we pulled out the same sequences from the same sewersheds week after week, which is arguably better than having split samples or technical replicates of the same nucleic acid extracts. One wouldn't expect to get jackpot amplification repeatedly, and only from a particular sewershed.

We have added a detailed explanation for how the cryptic lineages were identified and displayed.

**The analysis focuses almost entirely on the amino-acid mutations, although it tangentially mentions that the number of synonymous mutations is low. This analysis should be elaborated, including formal analysis of whether dN/dS exceeds 1 (the number of amino acid mutations exceeds the number of synonymous mutations more than expected for random nucleotide mutations).**

We tried to do this, but in many cases dS is zero. In addition to the dN/dS output in Fig. 7, we now explicitly display the synonymous changes in the Figs 1-6.

**Reviewer #2: 1) While the author’s use of the term ‘lineage’ is defined at the beginning of the results section and is useful for messaging, it is unclear how S-genotype based lineage designations do or don’t compare to whole-genome sequencing based lineage or clade designations. For example, is clade 20A different from 20G in this region? Or Alpha and Beta? It would be useful to have a SARS-CoV-2 phylogenetic tree built from just the sequenced region to illustrate the variants that can or cannot be cleanly differentiated by this method.**

**2) It is somewhat unclear how much diversity in these samples is detected that is attributed to clinically-derived specimens and known lineages. It would be particularly informative to generate a phylogenetic tree that visualizes all S-genotype diversity in a given wastewater specimen compared to a time-matched tree of the same region of S from clinical specimens sampled over a similar time frame. In theory, they should look very similar with the exception of the ‘cryptic lineages’ and doing such would provide statistical support for the existence of the unique lineage in the specimen. This wouldn’t need to be done exhaustively, but would add confidence to the analysis.**

This is a fair point and we explored using phylogenetic models to analyze the data. There was just too much convergent evolution to get meaningful output from phylogenetic trees. To address this comment, however, we added the changes in the major VOCs in the figures to provide a visual comparison of the cryptic lineages to known VOC lineages.

**3) The representations used for the S-genotypes in Figures 1-6 are redundant in showing results from assorted sewersheds and do not easily reflect the main points raised with each of the figures. Ideally, the sequences would be grouped on the y-axis by phylogenetic relatedness as opposed to by time. This could also provide statistical support for the somewhat arbitrarily defined ‘classes’ in Figure 4/5. The reference sequence should be more readily apparent at the top and the addition of common comparator sequences of VOCs could be added to make some points more salient (as done in Figure 7).**

We reorganized the figures as suggested.

**Reviewer #3: It is unclear to this reviewer how the authors handled resolving multiple SARS-CoV-2 lineages from mixed samples, which is harder using short-read sequence data.**

We added an explicit reference in the results to our published software pipeline that we developed for dealing with these complex samples.

**Page 32, lines 500-503. Although waste-water surveillance may contribute to SARS-CoV-2 epidemiology studies, the results described in the present manuscript may also show that some mutation constellations found in the environment apparently do not circulate between humans. The reason for that is still lacking. Therefore, it is hard to accept the statement “our results highlight the ability of wastewater-based epidemiology to more completely monitor SARS-CoV-2 transmission and genetic diversity than can patient based sampling”.**

This is a fair point. We removed the 'transmission' portion.

**Methods section: Three different protocols were used for water concentration and RNA extraction. Do the authors have a comparison on that? I mean, one of those had a better performance to recover viral RNA? How have these different protocols affected genome recovery and assembly?**

We do not. These are three independent groups doing wastewater surveillance and the focus of the collaboration was not to compare or standardize extraction techniques. We agree that subsequent work describing optimal sample preparation workflows would be broadly useful to the scientific community.

**Minor issues.**

**Reviewer #2: 1) While the authors report 9 sewersheds with cryptic lineages, it is unclear how many they performed surveillance on. In other words, it should be reported how many systems and specimens had no aberrant sequences.**

We added that this was 9 out of approximately 180 routinely monitored.

**3) The claims of where cryptic lineages derived from can really only be made in the context of a true phylogenetic comparison (i.e., lines 190-195, 217-219, etc).**

It won't be possible to perform a valid phylogenetic comparison without larger genome fragments, and this won't be possible without homogeneous samples. We have updated the wording to say the amino acid changes lineages are consistent with/not consistent with particular relatedness.

**4) A direct comparison of cryptic lineages would add substantial value. Some ad hoc comparisons are provided (i.e., lines 201-207), but it is really hard to try to compare them using the given representations.**

We switched the figures so that the two sewersheds with similar lineages are in the same figure so they can be more easily directly compared.

**5) Update the Omicron lineage comparison in lines 213-217 to account for new mutations in the sequenced S-gene region in sublineages BA.2-BA.5.**

Done.

**6) Reservoirs of SARS-CoV-2 in the gut have been suggested to be a contributor to long COVID. This may want to be added to the Discussion.**

We added an additional comment to the discussion about this possibility.

**Reviewer #3: How wastewater positivity correlates (spatially) with positive clinical cases in the present study?**

This was addressed in our previous study but was not within the scope of this study.

**It is unclear to this reviewer why the authors emphasize that “Many of the amino acid substitutions in these lineages occurred at residues also mutated in the Omicron variant of concern (VOC), often with the same substitution.” Do they suggest any relationship with Omicron emergence? Please clarify.**

"None of the wastewater lineages have combinations of amino acid changes consistent with having a common ancestor with Omicron and most were initially detected prior to the emergence of Omicron. However, these shared amino acid changes suggest that the cryptic lineages were under selective pressures similar to those that shaped the Omicron lineage."

**Do the authors have an explanation, or hypotheses, for cryptic lineages lasting for around six months without detection on human samples?**

Most likely long-term individual infections, as touched on in the discussion.

**Figure 1. Some of those positions that authors call “Omicron position” were detected before in other VOCs like Alpha, Beta, and Gamma. Thus, this reviewer suggests that the “Omicron position” is not the best option.**

We updated the Omicron designation to include BA.1, BA.2 and BA.5 as another reviewer requested. Because we've now included the PMs from the major VOCs it should be clearer to the reader what is meant by 'Omicron position'.

**Page 8, lines 151-152. Authors stated, “Amino acid changes similar to (green boxes) or identical to (orange boxes) changes in Omicron (BA.1) are indicated.” In fact, to Omicron VOC, but not all observed to the BA.1 lineage. Please see** [**https://outbreak.info/situation-reports?pango=BA.1**](https://outbreak.info/situation-reports?pango=BA.1)

We used the consensus sequences from the same page the reviewer lists. If the reviewer is referring to the N440K, the outbreak.info output is misleading. The amino acid change is fixed in all Omicron lineages (so far at least), but there have been so many sequences that had a dropout in that region that outbreak.info display indicates that the change is not fixed. It is. Not being able to detect an amino acid change because of a dropout is not the same as an amino acid change not being present.

**Page 19, lines 300-302. Authors stated: “This second sequence presumably represented a unique lineage that had not been detected by routine surveillance.” Since the USA has extensive genomics surveillance, authors should comment on the clinical surveillance specific in this area.**

We were referring to wastewater surveillance. This has been clarified.

**Page 19, lines 303-305. This elegant result further confirmed the VOC Alpha-related origin of the NY13 lineage.**

We are glad the reviewer agrees.

**Figure 7. I would suggest including the residue positions and the nucleotide positions according to the Wuhan RefSeq.**

That would make a figure that is already quite noisy even worse.

**Figure 7. How many samples underwent each sequencing protocol (e.g., Sanger or PacBio)? How many samples were analyzed for minor variants in the whole study? Was it enough for statistical analysis?**

There were not enough sequences to do statistical analyses. For most sites we only found one consensus sequence.

**Page 21, lines 341-343. Authors stated: “We speculate that the relative abundance of cryptic lineages was high because, during this period, NYC experienced the lowest levels of COVID-19 infections seen since the start of the pandemic.” Please, clarify this statement.**

We added a clarification to the sentence.

**Page 31, lines 480-482. What about probe-based genome capture? These protocols can generate complete genomes even with high Ct levels. Authors should comment on this.**

We believe that the reviewer is talking about patient samples, which are much more stable. We are unaware of anyone that can efficiently amplify long regions of SARS-CoV-2 from wastewater (2.5kb is currently the largest amplicon we have successfully amplified and sequenced using Pacific Biosciences instruments).

**Page 32, lines 506-508. Authors stated: “Importantly, patient sampling efforts, despite occurring with an intensity not seen in any prior epidemic, were unable to identify intermediary forms of most VOCs”.
That is not necessarily true. See the manuscript by Gräf and colleagues (**[**https://academic.oup.com/ve/article/7/2/veab091/6462077**](https://academic.oup.com/ve/article/7/2/veab091/6462077)**) about the origins of VOC Gamma.
Another critical point is that we now have much more data about mutations of concern that may aid in detecting a new VOC.**

We changed 'most' to 'many', though we think most is still accurate.
